# 

**Published:** 1869-09

**Authors:** 


### CONTENTS.

ORIGINAL COMMUNICATIONS.

Cleaning the Teeth ........ 361

The Hands ........ 364

Articulation ........ 365

Peculiar Dental Practice ........ 367

PROCEEDINGS OF SOCIETIES.

Official Proceedings of the Dental Convention, Atlanta, Georgia ........ 368

SELECTIONS.

On the Structure of Two Forms of Tooth Tumor ........ 372
Case of Tetanus Treated by Bromide of Potassium ........ 379
On the Therapeutic Action of Aconite and its Preparations ........ 382
De-Nicotinized Tobacco ........ 385
Taking Impressions for Partial Sets of Teeth ........ 386
Keeping Volatile Liquids ........ 388
A Test for Glycerin ........ 388
An Improved Battery ........ 388
Facial Neuralgia ........ 389
Cleft Palate ........ 389
Cessation of the Revista Medico Quirurjica y Dentistica ........ 389
Hare Lip ........ 390
Reproduction of Bone from Periosteum in Resection of Joints ........ 390
Artificial Respiration ........ 391
New Mode of Producing Oxygen ........ 391
Hydrogenium ........ 392

EDITORIAL.

American Dental Association ........ 399
In Toe-Toe ........ 400
Another Experience ........ 402
Found ........ 403
Southern Dental Association ........ 404
Personal ........ 404

